# The Impact of Cholecaciferol Supplementation on Bone Mineral Density in Long-Term Kidney Transplant Recipients

**DOI:** 10.3390/biom13040629

**Published:** 2023-03-31

**Authors:** Yuri Battaglia, Antonio Bellasi, Pasquale Esposito, Alessandra Bortoluzzi, Silverio Rotondi, Michele Andreucci, Fulvio Fiorini, Domenico Russo, Alda Storari

**Affiliations:** 1 Department of Medicine, University of Verona, 37129 Verona, Italy; 2 Nephrology and Dialysis Unit, Pederzoli Hospital, 37019 Verona, Italy; 3 Nephrology Unit, Ente Ospedaliero Cantonale, 6900 Lugano, Switzerland; 4 Division of Nephrology, Dialysis and Transplantation, University of Genoa, IRCCS Ospedale Policlinico San Martino, 16132 Genoa, Italy; 5 Rheumatology Unit, Department of Medical Sciences, University of Ferrara, 44124 Ferrara, Italy; 6 Department of Translational and Precision Medicine, Sapienza University of Rome, 00185 Roma, Italy; 7 Nephrology and Dialysis Unit, Department of Health Sciences, “Magna Graecia” University, 88100 Catanzaro, Italy; 8 Division of Nephrology and Dialysis, “Santa Maria della Misericordia” Hospital, 45100 Rovigo, Italy; 9 Department of Public Health, University Federico II, 80100 Napoli, Italy; 10 Nephrology and Dialysis Unit, St. Anna University Hospital, 44124 Ferrara, Italy

**Keywords:** bone mineral disease, vitamin D, kidney transplantation, Z-score, T-score, femoral neck, lumbar vertebral bodies, DEXA, KDIGO guidelines

## Abstract

Although reduced bone mineral density (BMD) is associated with a higher risk of fractures, morbidity, and mortality in kidney transplant patients (KTRs), there is no consensus on optimal treatment for the alterations of BMD in this population. This study aims at assessing the effect of cholecalciferol supplementation on BMD over a follow-up period of 2 years in a cohort of long-term KTRs. Patients with age ≥ 18 years were included and divided into two subgroups based on treatment with bisphosphonate and/or calcimimetics and/or active vitamin D sterols (KTRs-treated) or never treated with the above medications (KTRs-free). BMD was evaluated at lumbar vertebral bodies (LV) and right femoral neck (FN) with standard DEXA at the beginning and end of the study. According to World Health Organization (WHO) criteria, results were expressed as T-score and Z-score. Osteoporosis and osteopenia were defined as T score ≤ −2.5 SD and T score < −1 and >−2.5 SD, respectively. Cholecalciferol was supplemented at a dose of 25,000 IU/week over 12 weeks followed by 1500 IU/day. KTRs-free (*n*. 69) and KTRs-treated (*n*. 49) consecutive outpatients entered the study. KTRs-free were younger (*p* < 0.05), with a lower prevalence of diabetes (*p* < 0.05) and of osteopenia at FN (46.3 % vs. 61.2 %) compared to KTRs-treated. At the entry none of the study subjects had a sufficient level of cholecalciferol; Z-score and T-score at LV and FN were not different between groups. At the end of the study period, serum cholecalciferol concentration was significantly increased in both groups (*p* < 0.001); the KTRs-free group presented an improvement in both T-score and Z-score at LV (*p* < 0.05) as well as a lower prevalence of osteoporotic cases (21.7% vs. 15.9%); in contrast, no changes were recorded in KTR-treated individuals. In conclusion, supplementation with cholecalciferol ameliorated Z-score and T-score at LV in long-term KTRs who had been never treated with active or inactive vitamin D sterols, bisphosphonates, and calcimimetics. Future endeavours are needed to confirm these preliminary findings.

## 1. Introduction

Kidney transplant is the best replacement therapy for kidney function in patients with end-stage renal disease [1]. Although survival and quality of life after kidney transplantation have improved over the last few decades, kidney transplant recipients (KTRs) are susceptible to various complications, including cardiovascular risk, infections, cancer, post-transplant diabetes mellitus and bone disease [2].

Post transplantation bone disease (PTBD), characterized by disorders of calcium and phosphate, insufficiency or deficiency of vitamin D, secondary and tertiary hyperparathyroidism, osteodystrophy, osteoporosis, osteonecrosis, and bone fracture, occurs frequently in KTRs [3]. PTBD can result from the evolution of pre-existing Chronic Kidney Disease–Mineral and Bone Disorder (CKD–MBD) or the development of rapid bone loss in the post-transplant period [4]. It is caused by many factors such as corticosteroid dosage, immunosuppression therapy, graft dysfunction, fibroblast growth factor 23 (FGF-23) level, and ethnicity [5,6].

Vitamin D deficiency has been linked with low bone mineral density (BMD), a surrogate of bone mass, in KTRs [7]. Although ergocalciferol, cholecalciferol, and calcifediol supplementation effectively correct vitamin D deficiency or insufficiency, reduce parathyroid hormone (PTH), and improve calcium plasma levels, the effects of native sterols on BMD in KTRs remain undefined [8,9].

However, some studies have reported slight improvements in BMD with calcitriol [10,11]. Even though promising, the results with calcitriol were obtained in small groups of KTRs and after a follow-up period of no longer than one year [12].

Although several international guidelines recommend inactive vitamin D supplementation in chronic kidney disease (CKD) stages, their heterogeneous indications in terms of dose, modality of administration and target vitamin D level are often confounding [13,14]. In addition, no specific guideline concerning the replenishment of vitamin D deficiency in KTRs has been published. In the last revision of the Kidney Disease Improving Global Outcomes (KDIGO) guidelines on mineral metabolism management released in 2017 [15], therapeutic recommendations are only provided by the first 12 months from kidney transplantation; this was likely due to either absence of data from longer studies or conflicting results obtained with vitamin D supplementation.

Recently, we demonstrated that prolonged supplementation of 25-OH vitamin D did not modify BMD in long-term KTRs, despite a positive trend in the effect of 25-OH vitamin D on Z-score at the femoral neck (*p* = 0.056) [16], with 30% of the KTRs also receiving active vitamin D treatment.

Therefore, the purpose of the study is to further investigate the efficacy of vitamin D on BMD in long-term KTRs by assessing the differences in response to cholecalciferol supplementation between KTRs who have never received treatment with active vitamin D, bisphosphonate, or calcimimetics (KTRs-free), to those who have received such treatment (KTRs treated).

## 2. Materials and Methods

This single-centre observational, longitudinal study was carried out in KTRs who were followed up at the Nephrology Unit of Ferrara (Italy) University-Hospital from 2013 to 2021. Consecutive KTRs (≥18 years old) who had received a kidney from a cadaveric or living donor were recruited. KTRs with a history of bone fractures and parathyroidectomy were excluded. Patients were divided into two groups according to treatment with bisphosphonate and/or calcimimetics and/or vitamin D or never treated at study inception.

The research protocol obtained ethical approval from the Hospital Ethics Committee for Human Research (Code: 356) and written informed consent were collected. The procedures agreed with the Declaration of Helsinki.

By the year 2013, 25-OH-D assay and BMD examination entered in scheduled chemistry and procedure for KTRs referring to the Nephrology Unit. Based on 25-OH-D levels, vitamin D moderate (<30 ng/mL and ≥20 ng/mL), and severe insufficiency (<20 ng/mL and ≥10 ng/mL) or deficiency (<10 ng/mL) was corrected, using standard treatment strategy recommended for the general population [17,18]. Briefly, a course of 25,000 IU/week of cholecalciferol over 12 weeks was followed by 1500 IU/day.

Bone mineral density was measured via a Hologic Discovery DXA instrument (Hologic Inc., Waltham, MA, USA) with reported precisions ≤ 1% at the femoral neck (FN) and lumbar vertebral spine L1-L4 (LV). The machine was standardized by a standard phantom before each measurement. DEXA was evaluated when native vitamin D was prescribed, within a range of plus or minus two months, and a mean period of 24 months later, the BMD examination was repeated. According to the World Health Organization (WHO) criteria [19], DEXA results were expressed as T-score (standard deviation [SD] relative to young healthy adults), and Z-score (SD relative to age-matched controls). Normal bone density, osteopenia, and osteoporosis were defined as T score ≥ –1.0, T score < −1 and >−2.5 SD, and T score ≤ −2.5 SD at least in one site, respectively.

According to the DEXA results, the therapeutic treatment was adopted in accordance with the KDIGO clinical practice guidelines for the diagnosis, evaluation, prevention, and treatment of CKD-MBD 2009 [18].

Renal function was evaluated with the estimated glomerular filtration rate (e-GFR) according to the equation from the Modification of Diet in Renal Disease Study [20]. Body Mass Index (BMI) was calculated as weight (kg)/height^2^ (m^2^). Clinical characteristic and routine biochemistry, such as serum creatinine, serum calcium, serum phosphorus, intact PTH, 25-OH vitamin D, total protein, and albumin, were collected from digital patient archives. The biochemical parameters were determined using different methods, including immunoassay, enzymatic and colorimetric assay. Specifically, 25-OH vitamin D levels were measured using the immunoassays technique [21], whereas an intact PTH assay was employed to evaluate serum PTH levels [22].

### Statistical Analysis

Continuous variables were expressed as means and standard deviations (SD) or median and interquartile range (IQR) based on their distribution; and categorical variables as frequencies (percentage). The Student’s *t* test and chi-square test were used to compare continuous and categorical variables within as well as between groups. ANOVA was used to compare the laboratory and clinical differences among KTRs with normal BMD, osteopenia, or osteoporosis at LV and FN. Linear mixed model analysis [23] was used to test the impact of 25-OH-D use on Z-score and T-score changes (dependent variables) adjusted for sex, age, BMI, and presence of diabetes. Z-score and T-score changes were defined as Z-score and T-score at follow-up—Z-score and T-score at study inception. The same procedure was repeated for the femur and spine sites. SPSS software (version 28, IBM Corp., Armonk, NY, USA) was used for statistical analysis, and statistical significance was considered as *p* < 0.05.

## 3. Results

### 3.1. Baseline Study Population and DEXA Assessment

Data pertaining to 130 consecutive outpatients were collected and 12 were excluded (eight patients were excluded for the history of fractures and six patients for parathyroidectomy). Almost all patients (95.9%) received haemodialysis or peritoneal dialysis before renal transplant (KT); the remaining received KT pre-emptive. Glomerulonephritis (47.8%) and Autosomic Dominant Polycystic Kidney Disease (18.8%) were the primary causes of end-stage renal disease.

KTRs-free (*n*. 69) were mostly males (73.9%), middle-aged (mean age 52.20, SD 11.14 years), and no-smokers (89.9%). They also had a longer transplant vintage (median 88.0, interquartile range 28.0–188.5 months). Females (83.3%) were in their post-menopause stage.

KTRs-treated (*n*. 49) were older (*p* < 0.05) with a higher prevalence of diabetes (20.4% vs. 7.2%; X^2^ 1; N = 118; *p* < 0.05) (Table 1). Bisphosphonate (*n*. 11) and/or calcimimetics (*n*. 11) and/or calcitriol (*n*. 34) were used in KTRs-treated. Both groups had insufficient to deficient levels of 25-OH-D and none of the entire cohort had sufficient levels of 25-OH-D. Consequently, inactive vitamin D supplementation was started in all 69 KTRs-free patients and 49 KTRs-treated patients.

Osteopenia and osteoporosis were recorded in 27 (39.1%) and 15 (21.7%) KTRs-free at LV; as well as in 32 (47.8%) and 10 (14.9%) KTRs-free at FN, respectively. Compared with KTRs-free, a higher prevalence of osteopenic cases at FN (61.2%) was found among KTRs-treated.

No differences were found in DEXA parameters (Table 2), biochemistry and clinical characteristics, and immunosuppressive therapy whatever the BMD was (normal, osteopenia, or osteoporosis) in both KTRs groups.

### 3.2. Follow-Up Study Population and DEXA Assessment

At the end of follow-up (mean duration 27.7, SD 3.4 months) in both groups (89.8% of KTRs-treated and 88.1% of KTRs-free) cholecalciferol levels of at least 20 mg/dL were reached. A significantly increased concentration of calcium (t −2.74; df 135; *p* < 0.05), 25-OH-D vitamin (t −9.42; df 129; *p* < 0.001), and eGFR (t 2.05; df 132; *p* < 0.05), was found in KTRs-free; only 25-OH-D vitamin concentration increased in KTRs-treated group (t −9.22; df 95; *p* < 0.001). Upon stratification according to the WHO classification at the follow-up assessment, no significant difference was observed between the two groups, except for higher vitamin D levels in the KTRs-treated group with osteoporosis compared to the KTRs-free group (*p* = 0.03) (Table 3).

None of the KTRs-free patients had started therapy with bisphosphonates and/or calcimimetics, while three KTRs-treated patients began treatment with calcimimetics, and none of the KTRs-treated patients had discontinued bisphosphonate therapy. Calcium supplementation was not administrated to any patients in either group during the follow-up period. There was only one recorded episode of chronic kidney rejection in each group, but no graft loss occurred. Compared to the baseline assessment, no significant difference in eGFR was found for both groups. Notably, three KTRs-treated and one KTR-free had an eGFR greater than 90 mL/min, but only one KTRs-treated developed chronic kidney disease.

At the time of the repeated DEXA examination, the prevalence of osteoporosis in the femoral neck (18.3%) and lumbar vertebrae (15.9%) was lower in the KTRs-treated and KTRs-free groups, respectively (Table 4).

Among KTRs-free, a positive interaction of inactive vitamin D supplementation with Z score and T score change at lumbar vertebral bodies (*p* < 0.05) was found in linear mixed model analysis (Table 5).

On the other hand, no significant 25-OH-D effect on T score, Z score, and BMD variations at the femoral neck was observed. Similarly, among KTRs-treated, no impact of inactive vitamin D on changes in DEXA parameters was found.

## 4. Discussion

This study suggests that cholecalciferol supplementation improves Z-score and T-score of LV in long-term KTRs-free. The positive effects are long-lasting (up to mean 27.7; SD 3.4 months).

Prevalence of osteopenia or osteoporosis either at LV and at FN in the baseline DEXA was not different between KTRs-treated (81.6%) and KTRs-free (62.7%). The percentage observed in KTRs-treated is in line with that reported by others in long-term KTRs chronically treated with vitamin D supplements or with bisphosphonates in whom the prevalence of osteoporosis and osteopenia was up to 80%.

In the present study, it might have been expected to find a higher percentage of osteoporosis and osteopenia in patients KTRs-free compared to KTRs-treated. However, this was not observed. One possible explanation for this discrepancy in baseline DEXA assessments could be the clinical and demographic characteristics of KTRs-free and KTRs-treated. Specifically, KTRs-treated were significantly older (56.6 vs. 52.2 years) and most were diabetics (20.4 % vs. 7.4%)

Supplementation increased the serum concentration of cholecalciferol in almost all patients and improved Z-score and T-score at LV. However, the improvement was not significantly different compared to the baseline value; nevertheless, there was a notable decrease in the percentage of osteoporotic patients (21.7% vs. 15.9%). Ameliorative effects of cholecalciferol therapy have been reported [24,25]. Indeed, a 4000 IU daily dose of cholecalciferol significantly increased T-score at LV in KTRs with osteoporosis/osteopenia after a median observation period of only 12 months after kidney transplant [26]. The annual dose reported in that study is comparatively similar to that of the present study. Altogether these findings seem to indicate that cholecalciferol therapy is effective at LV regardless of transplant vintage [27,28,29].

The mechanisms underlying the positive cholecalciferol effect on BMD in KTRs are still largely unknown. However, the data from the present study suggest that cholecalciferol therapy does not improve BMD in KTRs who are already receiving treatment with active vitamin D and/or bisphosphonates. Similarly, our recent study, which analyzed a partially similar cohort of long-term KTRs not receiving bisphosphonates and/or calcimimetics, but receiving treatment with or without active vitamin D, demonstrated that 25-OH vitamin D supplementation did not modify Z-score, T-score, and BMD.

In contrast with these findings, a network meta-analysis showed that the combined therapy of bisphosphonate, with calcium and vitamin D analogs, improved BMD at LV and FN. However, when the authors performed an indirect comparison of bisphosphonate plus calcium use with or without vitamin D analogue supplementation, no difference in BMD was found [30].

The findings observed in the present study seem to indicate that the efficacy of cholecalciferol in improving BMD status is hampered in KTRs under therapy with bisphosphonates and/or active vitamin D. It can be hypothesized that administration of bisphosphonates and/or active vitamin D, may inhibit the bone loss and increase BMD of cortical and trabecular bone by suppressing the osteoclast activity [31,32].

However, the study has several limitations. Firstly, patients were not randomized, and relevant biochemical markers, such as FGF-23 [33,34,35], and ultrasound data [36,37,38] were not available to better characterize the population. Secondly, no data were collected on clinical factors, such as lifestyle, skeletal muscle status [39] and level of physical activity [40,41,42], which are known to be involved in BMD changes. Thirdly, all patients in treatment with bisphosphonates, active vitamin D, and calcimimetics were allocated to the KTRs-treated groups, even though the individual o combined effect of these drugs on BMD may vary. Furthermore, the limited sample size did not permit to stratify the patients based on the type of treatment received and to adjust the linear mixed model for other variables such as PTH, phosphorus levels, type of immunosuppression, and corticosteroid doses. Finally, no previous BMD values were available for the KTRs-treated group.

## 5. Conclusions

This study suggests that cholecalciferol supplementation ameliorates Z-score and T-score at lumbar vertebral bodies in long-term KTRs who had never received active or inactive vitamin D sterols, bisphosphonates, and calcimimetics. While it is mandatory to achieve sufficient levels of 25-OH vitamin D regardless of the transplant vintage, cholecalciferol supplementation may be more effective at improving osteoporosis as assessed by DEXA, in long-term KTRs not receiving bisphosphonate and/or active vitamin D treatment. Further multicentre randomized control trials are required to confirm the positive effects of cholecalciferol on BMD or evaluate the impact on clinical outcomes in long-term KTRs.

## Figures and Tables

**Table 1:** Demographic and biochemical data of KTRs-treated and KTRs-free at baseline.

	KTRs-Free (*n.* 69)		KTRs-Treated (*n.* 49)		*p*-Value
Age, years *	52.20	(11.14)	56.61	(12.32)	0.04 °
Male, *n* (%)	51	(73.91)	30	(61.22)	0.14
Smoker, *n* (%)	7	(10.14)	4	(8.16)	0.71
Diabetes, *n* (%)	5	(7.24)	10	(20.40)	0.04 °
BMI, kg/m^2^ *	24.71	(2.97)	23.29	(3.94)	0.21
HD Vintage Pre-KT, months *	28.14	(30.32)	28.29	(21.51)	0.97
KT pre-emptive, *n* (%)	5	(7.24)	2	(4.08)	0.69
KT duration, months **	88	(28–188)	61	(21–154)	0.50
Systolic BP, mmHg *	130.00	(14.30)	128.47	(15.28)	0.57
Diastolic BP, mmHg *	77.83	(7.35)	77.76	(8.96)	0.96
FC, bpm *	73.80	(11.41)	71.88	(10.50)	0.35
serum Creatinine, mg/dL *	1.43	(0.55)	1.30	(0.44)	0.17
eGFR, ml/min *	53.84	(16.87)	56.12	(18.69)	0.49
serum Calcium, mg/dL *	9.40	(0.43)	9.39	(0.53)	0.98
serum Phosphorus, mg/dL *	3.21	(0.60)	3.20	(0.71)	0.92
25-OH Vitamin D, ng/mL *	15.43	(8.07)	14.70	(9.29)	0.64
intact PTH, pg/mL *	103.38	(80.01)	108.96	(68.50)	0.69
Total Protein, g/dL *	6.61	(0.52)	6.62	(0.60)	0.91
Albumin, % *	58.88	(4.14)	58.51	(4.82)	0.65
Immunosuppressive Therapy					
Steroids, *n* (%)	63	(91.30)	45	(91.83)	0.71
Steroids dose, mg *	3.45	(1.35)	3.82	(1.19)	0.12
Cyclosporine, *n* (%)	28	(40.57)	19	(38.77)	0.74
Tacrolimus, *n* (%)	37	(53.62)	28	(57.14)	0.84
Mycophenolate mofetil, *n* (%)	44	(63.76)	32	(65.30)	0.96
Azathioprine, *n* (%)	8	(11.59)	6	(12.24)	0.96
mTOR inhibitors, *n* (%)	4	(5.79)	7	(14.28)	0.13

* Data expressed as Mean (Standard Deviation); ** Median (Inter Quartile Range); ° statistically significance; BP: Blood Pressure; BMI: Body Mass Index; eGFR: estimate Glomerular Filtrate Rate; HD: Haemodialysis; PTH: Parathormone; KT: Kidney Transplant.

**Table 2:** DEXA parameters evaluated at the right femoral neck (FN) and lumbar vertebral bodies (LV) according to WHO Classification in KTRs-free and KTRs-treated at baseline.

WHO	FN	Mean	SD	LV	Mean	SD
Normal BMD	T score KTRs-free	−0.572	0.698	T score KTRs-free	0.137	0.954
	T score KTRs-treated	−0.100	0.676	T score KTRs-treated	−0.312	0.527
	*p*-value	0.89		*p*-value	0.84	
	Z score KTRs-free	0.112	0.724	Z score KTRs-free	0.731	1.136
	Z score KTRs-treated	0.700	0.880	Z score KTRs-treated	0.476	0.631
	*p*-value	0.57		*p*-value	0.40	
Osteopenia	T score KTRs-free	−1.659	0.459	T score KTRs-free	−1.670	0.322
	T score KTRs-treated	−1.773	0.371	T score KTRs-treated	−1.737	0.434
	*p*-value	0.28		*p*-value	0.55	
	Z score KTRs-free	−0.834	0.524	Z score KTRs-free	−1.015	0.541
	Z score KTRs-treated	−0.763	0.629	Z score KTRs-treated	−1.284	1.613
	*p*-value	0.63		*p*-value	0.42	
Osteoporosis	T score KTRs-free	−2.590	0.242	T score KTRs-free	−3.100	0.425
	T score KTRs-treated	−2.690	0.600	T score KTRs-treated	−3.190	0.546
	*p*-value	0.63		*p*-value	0.64	
	Z score KTRs-free	−1.500	0.419	Z score KTRs-free	−2.333	0.530
	Z score KTRs-treated	−1.650	0.843	Z score KTRs-treated	−2.220	0.989
	*p*-value	0.62		*p*-value	0.74	

BMD: Bone Mineral Density; KTRs: Kidney Transplant Recipients; SD: Standard Deviation; WHO: World Health Organization.

**Table 3:** Biochemical data at the time of the second assessment for both groups.

Sites	Variables	WHO Classification	KTRs Free	KTRs Treated	*p* °°	Sites	Variables	WHO Classification	KTRs Free	KTRs Treated	*p* °°
FN	Calcium mg/dL *	Normal BMD	9.59 (0.50)	9.27 (0.48)	0.92	LV	Calcium mg/dL *	Normal BMD	9.55 (0.42)	9.46 (0.45)	0.49
		Osteopenia	9.63 (0.47)	(9.50) (0.50)	0.27			Osteopenia	9.70 (0.44)	9.45 (0.59)	0.11
		Osteoporosis	9.41 (0.68)	9.44 (0.72)	0.92			Osteoporosis	9.41 (0.79)	9.44 (0.59)	0.94
		Statistics °	0.48	0.50				Statistics °	0.27	0.99	
FN	Phosphorus mg/dL *	Normal BMD	3.29 (0.58)	3.37 (0.68)	0.70	LV	Phosphorus mg/dL *	Normal BMD	3.38 (0.60)	3.31 (0.63)	0.67
		Osteopenia	3.37 (0.59)	3.24 (0.50)	0.35			Osteopenia	3.30 (0.58)	3.40 (0.55)	0.54
		Osteoporosis	3.42 (0.51)	3.26 (0.74)	0.57			Osteoporosis	3.35 (0.48)	3.08 (0.51)	0.21
		Statistics °	0.79	0.82				Statistics °	0.85	0.36	
FN	25-OH Vit D ng/mL *	Normal BMD	30.47 (8.66)	27.10 (8.25)	0.29	LV	25-OH Vit D ng/mL *	Normal BMD	27.81 (8.92)	28.89 (9.54)	0.69
		Osteopenia	28.49 (9.82)	32.71 (10.44)	0.10			Osteopenia	30.69 (9.25)	30.29 (8.88)	0.88
		Osteoporosis	30.80 (5.85)	33.40 (5.67)	0.34			Osteoporosis	29.84 (7.58)	37.79 (8.63)	0.03 **
		Statistics °	0.64	0.20				Statistics °	0.48	0.04 **	
FN	iPTH pg/mL *	Normal BMD	81.89 (37.93)	81.07 (27.09)	0.95	LV	iPTH pg/mL *	Normal BMD	82.18 (41.91)	90.00 (41.11)	0.53
		Osteopenia	82.08 (40.85)	89.90 (42.03)	0.46			Osteopenia	77.50 (32.39)	88.46 (32.67)	0.26
		Osteoporosis	95.96 (41.21)	92.78 (38.92)	0.86			Osteoporosis	105.85 (43.61)	86.10 (44.00)	0.31
		Statistics °	0.59	0.79				Statistics °	0.12	0.96	

* Data expressed as Mean (Standard Deviation); ** statistically significance; ° ANOVA test; °° Student’s *t* test; BMD Bone Mineral Density; FN: Femoral Neck; LV: Lumbar Vertebral spine; iPTH: intact Parathormone.

**Table 4:** T-score, Z-score, and BMD gains at 2 to 3 years follow-up in untreated and treated bisphosphonate and/or calcimimetics and/or active vitamin D patients.

	Gain T Score				Gain Z Score				Gain BMD			
	Lumbar Spine	*p*	Femoral Neck	*p*	Lumbar Spine	*p*	Femoral Neck	*p*	Lumbar Spine	*p*	Femoral Neck	*p*
KTRs-free	0.03 ± 0.51	0.88	−0.07 ± 0.35	0.82	0.07 ± 0.46	0.92	−0.03 ± 0.47	0.68	−0.03 ± 0.27	0.30	0.01 ± 0.07	0.66
KTRs-treated	0.01 ± 0.45	0.75	−0.04 ± 0.28	0.66	0.07 ± 0.44	0.35	0.02 ± 0.28	0.98	−0.01 ± 0.04	0.77	0.01 ± 0.05	0.98
*p*-value	0.77		0.64		0.95		0.41		0.47		0.51	

Data are expressed as Mean Standard ± Deviation; BMD: Bone Mineral Density; KTRs: Kidney Transplant Recipients.

**Table 5:** Mixed model effect of inactive vitamin D on T-score and Z-score at lumbar vertebral bodies among kidney transplant patients not in treatment with active vitamin D and/or bisphosphonate and/or calcimimetics.

Parameter	Estimate	Std. Error	df	t	Sig.	95% CI	
						LB	UB
Intercept	−2.449	1.115	121.817	−2.196	0.030	−4.658	−0.241
Age	−0.003	0.013	83.970	−0.278	0.781	−0.030	0.023
Sex	−0.947	0.344	68.559	−2.749	0.008 *	−1.635	−0.259
BMI	0.102	0.037	131.997	2.719	0.007 *	0.028	0.177
25-OH-Vit D	0.007	0.003	80.521	2.045	0.044 *	0.001	0.014
Diabetes	1.284	0.581	68.863	2.211	0.030 *	0.125	2.444
A. Dependent Variable: Z Score at lumbar vertebral bodies							
Intercept	−2.205	1.149	117.142	−1.918	0.057	−4.481	0.071
Age	−0.038	0.013	81.308	−2.826	0.006 *	−0.064	−0.011
Sex	−0.310	0.341	69.286	−0.911	0.366	−0.990	0.369
BMI	0.119	0.039	130.519	3.004	0.003 *	0.040	0.197
25-OH-Vit D	0.008	0.003	80.949	2.063	0.042 *	0.001	0.015
Diabetes	1.288	0.575	69.825	2.240	0.028 *	0.141	2.435
B. Dependent Variable: T Score at lumbar vertebral bodies							

* Statistically significance; BMI: Body Mass Index; CI: Confidence Interval; LB: Lower Bound; UB: Upper Bound.

## Data Availability

Not available.
